# Evaluation of a Decentralized Donor-Derived Cell-Free DNA Assay for Kidney Allograft Rejection Monitoring

**DOI:** 10.3389/ti.2024.13919

**Published:** 2024-12-17

**Authors:** Alexandre Loupy, Anaïs Certain, Narin S. Tangprasertchai, Maud Racapé, Cindy Ursule-Dufait, Kawthar Benbadi, Marc Raynaud, Evgeniya Vaskova, Corina Marchis, Sílvia Casas, Tim Hague, Oriol Bestard, Delphine Kervella, Carmen Lefaucheur, Thierry Viard, Olivier Aubert

**Affiliations:** ^1^ Université Paris Cité, Institut national de la santé et de la recherche médicale (INSERM) U970, Paris Institute for Transplantation and Organ Regeneration PITOR, Paris, France; ^2^ Department of Kidney Transplantation, Necker Hospital, Assistance Publique - Hôpitaux de Paris, Paris, France; ^3^ CareDx, Brisbane, CA, United States; ^4^ Department of Nephrology and Kidney Transplantation, Vall d’Hebron University Hospital, Vall d’ Hebrón Research Institute, Vall d’ Hebrón Barcelona Campus Hospital, Barcelona Autonomous University, Barcelona, Spain; ^5^ Kidney Transplant Department, Saint-Louis Hospital, Assistance Publique - Hôpitaux de Paris, Paris, France

**Keywords:** AlloSeq, dd-cfDNA, liquid biopsy, allograft rejection, non-invasive diagnosis

## Abstract

Donor-derived cell-free DNA (dd-cfDNA) is an emerging non-invasive biomarker for allograft injury detection. This study aimed to evaluate a new, decentralized dd-cfDNA testing kit against a centralized dd-cfDNA testing service broadly utilized in the United States. Kidney transplant recipients with decentralized and centralized dd-cfDNA measurements and concomitant kidney allograft biopsies were included in the study. 580 kidney allograft recipients from 3 referral centers were included for 603 total evaluations. Correlation between assays was evaluated using r-squared (*r* ^2^) and Spearman’s rank correlation test, and associations with rejection using logistic regression analyses and discrimination using area under the curve. Mean dd-cfDNA levels from decentralized and centralized tests were 0.51% ± 0.81% and 0.43% ± 0.78%, respectively. The assays were highly correlated, with *r* ^2^ = 0.95 and Spearman’s rank correlation 0.88 (*p* < 0.0001). Both tests showed significant association with allograft rejection (*p* < 0.0001) and good and similar discriminations to predict rejection (AUC: 0.758 for the decentralized and AUC: 0.760 for the centralized dd-cfDNA; *p* = 0.8466). Consistency between the assays was also confirmed across clinical scenarios including post-transplant timepoint, allograft stability, and allograft rejection subcategories. This decentralized dd-cfDNA assessment demonstrates high accuracy and value to non-invasively monitor kidney recipients.

## Graphical Abstract

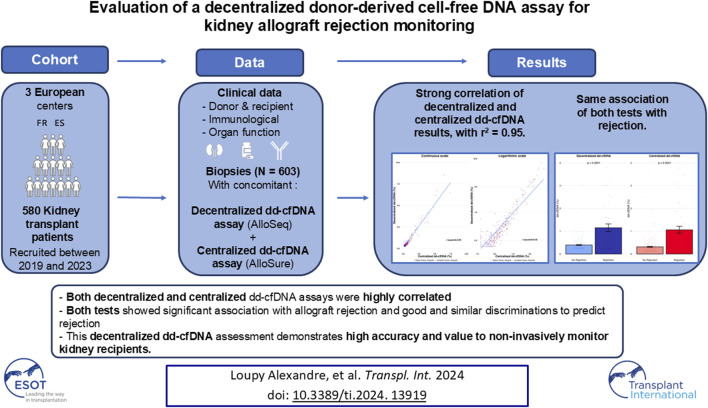

## Introduction

Allograft rejection remains the main cause of allograft loss after transplantation with detrimental consequences in terms of mortality, morbidity, and quality of life [1]. The gold standard to diagnose allograft rejection relies on tissue biopsy which is an invasive, costly procedure with potential pitfalls for interpretation [2, 3]. Measurement of various biomarkers offers non-invasive alternatives to the traditional biopsy with lower risk to the patient, less subjectivity, and greater convenience and flexibility. Serum creatinine (SC) and donor-specific antibodies (DSA) have been identified as informative biomarkers for kidney transplant monitoring, but SC has low sensitivity and specificity [4].

Donor-derived cell-free DNA (dd-cfDNA) has emerged as a clinically relevant biomarker in solid organ transplantation [5–8], and has been increasingly characterized with kidney transplant patients [9–13]. cfDNA naturally circulates in the bloodstream as a result of normal cell death mechanisms and can be influenced by factors like metabolic processes or overall health [14]. After transplantation, the allograft releases cfDNA, characterized as dd-cfDNA, into the recipient’s blood stream at low levels, which increases in cases of injury, rejection, or other malfunction [9–12]. Genetically distinct from the recipient’s own cfDNA (i.e., excluding cases where donor and recipient are monozygotic twins), dd-cfDNA can be detected and quantified at relatively low levels, making it an effective biomarker for routine post-transplant surveillance.

The American Society of Transplant Surgeons (ASTS) has recently recommended dd-cfDNA testing in adult kidney transplant recipients to monitor for rejection as a critical component of post-transplant surveillance [15], while the European Society for Organ Transplantation (ESOT) has advocated for the unmet need for non-invasive patient monitoring, the potential of dd-cfDNA in early detection of rejection, and its role in clinical decision-making [16]. Following those recommendations, centralized dd-cfDNA testing platforms are widely utilized by transplant physicians for routine post-transplant monitoring. Patient specimens are shipped from clinics and hospitals, dd-cfDNA testing is performed at the manufacturer’s appropriately certified and accredited laboratory, and results are released to clinicians. Until recently, dd-cfDNA testing was primarily available through such centralized testing services [17]. However, the possibility of decentralized dd-cfDNA testing solutions hold key advantages including the convenience of onsite laboratory testing, enabling broader access and adoption, and enhanced flexibility and agility with clinical decisions.

The decentralized dd-cfDNA assay of focus in this study is a commercially available, CE-IVDD testing kit utilizing polymerase chain reaction (PCR) amplification of a proprietary single nucleotide polymorphism (SNP) panel and next-generation sequencing (NGS) technology to provide relative dd-cfDNA quantification for solid organ transplant recipients, without requiring genotyping. This assay offers up to 24-sample throughput with turnaround time of 24 h from cfDNA sample to dd-cfDNA result (CareDx Instructions for Use: AlloSeq cfDNA Assay Instructions for Use IFU090 Version 6.0. Brisbane, CA) [18].

While decentralized dd-cfDNA testing technology opens avenues for onsite testing to facilitate therapeutic decisions for patient care [19], the direct comparison of its performance with respect to a well-characterized centralized dd-cfDNA testing platform [10, 20] and accuracy of clinical rejection detection have not previously been assessed on a single cohort. To address this timely issue, this large-scale, multicenter evaluation of a decentralized dd-cfDNA assay for kidney transplant patient monitoring was performed to assess both accuracy in comparison with a commonly used centralized dd-cfDNA testing platform and capacity to detect kidney allograft rejection.

## Materials and Methods

### Study Design and Population

This large, multicenter trial included 580 kidney transplant patients from 3 centers. Patients were prospectively recruited in Necker Hospital, Paris, France; Saint-Louis Hospital, Paris, France; and Vall d’Hebrón University Hospital, Barcelona, Spain, between May 2019 and July 2023. Patients with combined organ transplantation or patient that had received a previous solid organ transplant (other than a kidney), pregnant women, recipients of a graft from a monozygotic twin, and patients who had received a bone marrow transplant were excluded. 792 samples with AlloSeq results were screened in the study. Evaluation without concomitant biopsies (n = 102, 12.9%) or AlloSure results (n = 87, 11.0%) were excluded. A total of 603 evaluation with AlloSeq and AlloSure results with a concomitant biopsy were included. All data were anonymised and prospectively entered at the time of transplantation, and were updated at several timepoints (3, 6, and 12 months post transplantation and annually thereafter), and at each clinical event using a standardised protocol to ensure harmonisation across study centers. Data were submitted for an annual audit to ensure data quality. Data were retrieved from the database in November 2023. The study was approved by the institutional review board of the Paris Transplant Institute for participating centers and was conducted in accordance with the Declaration of Helsinki. All patients provided written informed consent at the time of transplantation.

In the transplant referral center Vall d’Hebrón University Hospital, data were collected as part of routine clinical practice and entered in each center’s database in compliance with local and national regulatory requirements and sent anonymised to the Paris Transplant Group.

### Data Collection

Clinical data covered demographic parameters, including recipient age, sex, and transplant characteristics; biological parameters, including kidney allograft function, proteinuria, and anti-HLA DSA specificities and levels; and allograft pathology data, including Banff lesion scores and diagnoses. Kidney allograft function was assessed by the glomerular filtration rate estimated by the Modification of Diet in Renal Disease Study equation (eGFR) and proteinuria level using the protein/creatinine ratio. The presence of circulating DSA against HLA-A, HLA-B, HLA-Cw, HLA-DR, HLA-DQ and HLA-DP was assessed using single-antigen flow bead assays (One Lambda, Inc., Canoga Park, CA, United States) on a Luminex platform [21] at the time of dd-cfDNA evaluation. Beads with a normalized mean fluorescence intensity (MFI), a measure of donor-specific antibody strength, of greater than 500 units were judged as positive, as described previously [22]. HLA typing of the transplant recipients and donors was performed using an Innolipa HLA Typing Kit (Innogenetics, Ghent, Belgium). Allograft biopsies performed at the time of dd-cfDNA measurement were scored and graded from 0 to 3 according to the Banff 2019 classification [23] for allograft pathology for the following histological factors: glomerular inflammation (glomerulitis), tubular inflammation (tubulitis), interstitial inflammation, endarteritis, peritubular capillary inflammation (capillaritis), transplant glomerulopathy, interstitial fibrosis, tubular atrophy, arteriolar hyalinosis and arteriosclerosis. Additional diagnoses provided by the biopsy (e.g., the diagnoses of primary disease recurrence, BK virus nephropathy) were recorded. The biopsy sections (4 μm) were stained with periodic acid-Schiff, Masson’s trichrome, and hematoxylin and eosin. C4d staining was performed via immunohistochemical analysis on paraffin sections using polyclonal human anti-C4d antibodies.

### Circulating Nucleic Acid Extraction

Whole blood was drawn into Cell-Free DNA BCT (Streck, La Vista, NE, United States, 218997) following manufacturer instructions for use (IFU). Plasma was isolated within 7 days of blood draw according to Streck Double Spin Protocol 2. Filled blood tubes were centrifuged for 10 min at 1,600 × g at room temperature, the upper plasma layer was transferred to a new conical tube then centrifuged for 10 min at 16,000 × g at room temperature, and clarified plasma was transferred to a new, appropriately labeled screw-top tube for storage at −80°C. cfDNA extraction was performed with QIAamp Circulating Nucleic Acid Kit (Qiagen, Hilden, Germany, 55114) following the manufacturer protocol Purification of Circulating Nucleic Acids from 4 mL Plasma, using 25 µL Buffer AVE for elution. Purified cfDNA was stored at −80°C and shipped on dry ice with temperature monitors. Aliquots of the same cfDNA sample were used to perform centralized and decentralized dd-cfDNA tests.

### Decentralized dd-cfDNA Measurement

AlloSeq cfDNA (CareDx, Brisbane, CA, United States) was performed following manufacturer IFU (CareDx Instructions for Use: AlloSeq cfDNA Assay Instructions for Use IFU090 Version 6.0. Brisbane, CA). Purified cfDNA samples were normalized to 0.625 ng/uL for 10 ng input in 16 µL. Multiplex PCR was performed to simultaneously amplify and index 202 SNP regions with the designated thermocycling protocol. The resulting PCR products were pooled with a fixed volume, then cleaned using the specified magnetic bead purification protocol. The final cleaned library pool was quantified via Qubit dsDNA Quantification High Sensitivity (Thermo Fisher, Waltham, MA, United States, Q32851), then diluted and denatured for paired-end read sequencing on MiSeq (Illumina, San Diego, CA, United States) using MiSeq Reagent Kit v3, 150-cycle (Illumina, MS-102-3001). After each sequencing run, two post-run washes were performed to prevent index contamination between runs, the first using diluted bleach with the template line wash, and the second using detergent only without the template line wash.

Recipient and sample information, including genetic donor-recipient relationship, were entered into the AlloSeq cfDNA Software version 2.2.0 (CareDx), and FASTQ files generated from Illumina Real-Time Analysis software base calls were supplied following manufacturer IFU (CareDx Instructions for Use: AlloSeq cfDNA Software Instructions for Use IFU091 Version 6.0. Brisbane, CA). The proprietary data analysis algorithm, described below, automatically analyzed sequencing reads for each SNP region to determine the relative fraction of donor and recipient DNA in each sample. Target loci include 202 SNPs with genome-wide distribution (see Supplementary Table S1), multiethnic representation, high uniformity, and sufficient coverage to distinguish even genetically related donor-recipient pairs.

Calculation of dd-cfDNA was performed automatically within the AlloSeq cfDNA Software. Illumina short reads were trimmed for low quality ends and sequencing adaptors, then aligned to a custom reference assembly, containing the two expected alleles for each biallelic SNP, using a custom Needleman-Wunsch short read alignment algorithm. Mappings with both reads aligned were retained and the proportion of nucleotide signals at each targeted SNP locus were calculated. SNP results with low coverage or multiallelic (>2 allele) calls were excluded. Minor signals at homozygous SNPs were assumed as the dd-cfDNA fraction. Heterozygous SNP positions were excluded using a 30%–70% minor signal filter (only in cases where genotypes are not provided), and unexpected SNP results were assumed to be background noise and filtered out. Mean and standard deviation were calculated for the remaining minor signals, and any statistical outliers in the dataset were removed via z-score outlier detection (eliminating imbalanced heterozygous SNPs outside the 30–70 range, which could occur due to primer site differences), and mean and standard deviation recalculated, if necessary. The mean was adjusted for the expected 1:2:1 ratio (identical homozygous : heterozygous : opposite homozygous) of biallelic SNPs and for any genetic relatedness between recipient and donor, then reported as the final dd-cfDNA fraction in the software interface. Fully transparent visualization of results at every SNP locus and multiple QC metrics (too many outliers, markers passing filter, uniformity, average marker coverage, and total reads) were also reported.

Genotyping is not required for this assay, but recommended in cases of dd-cfDNA greater than approximately 30%, which are not clinically likely in cases of kidney transplant (CareDx Instructions for Use: AlloSeq Software Instructions for Use IFU091 Version 6.0. Brisbane, CA). Values in that high range would be reported as calculated by the AlloSeq cfDNA Software algorithm, but would only be considered accurate with one or more associated genotypes. In the absence of recipient and/or donor genotype(s), the minor fraction is automatically assigned to the donor (CareDx Instructions for Use: AlloSeq cfDNA Assay Instructions for Use IFU090 Version 6.0. Brisbane, CA; CareDx Instructions for Use: AlloSeq Software Instructions for Use IFU091 Version 6.0. Brisbane, CA).

### Centralized dd-cfDNA Measurement

AlloSure Kidney (CareDx) was performed by trained CareDx R&D staff, following the same protocols as the CareDx CLIA-certified, CAP-accredited laboratory, as described previously [11, 24]. Purified cfDNA samples were used with fixed-volume input only and amplified via targeted PCR with primers for 405 SNP regions. Intermediate PCR products were cleaned with magnetic beads and indexing performed via another PCR with sequencing indexes and adapters. Resulting PCR products were pooled and cleaned with magnetic beads. The final cleaned library pool was quantified via Qubit dsDNA Quantification High Sensitivity (Thermo Fisher, Q32851), then diluted for single read sequencing on NextSeq (Illumina) using NextSeq 500/550 Mid Output Kit v2.5, 150 cycles (Illumina, 20024904) or NextSeq 500/550 High Output Kit v2.5, 150 cycles (Illumina, 20024907), depending on sample throughput. Analysis pipeline and procedures were described previously [11, 24].

### Statistical Analysis

Continuous variables were described using means and standard deviations or medians and interquartile ranges (IQR). Means and proportions were compared between groups using Student’s t-test, analysis of variance (or Mann Whitney test if appropriate), or the Chi squared test (or Fisher’s exact test if appropriate). The correlations between the decentralized and centralized dd-cfDNA results were assessed using the r-squared metric (*r* ^2^) and Spearman’s rank correlation test. The associations of decentralized and centralized dd-cfDNA with rejection were assessed using logistic regression analyses. The discrimination ability was evaluated using area under the curve (AUC) [25]. All analyses were performed using R (version 4.1.2, R Foundation for Statistical Computing) and STATA (version 17). Values of *p* < 0.05 were considered significant, and all tests were two-tailed.

## Results

### Characteristics of Patients at Baseline and at the Time of dd-cfDNA Measurement

The kidney transplant cohort was comprised of 580 patients and 603 evaluations with measurement of circulating dd-cfDNA post-transplant at the time of allograft biopsy. The mean recipient age was 51.35 ± 16.63 years. The mean donor age was 53.61 ± 17.50 years. A total of 399 (68.79%) patients received a kidney from a deceased donor, while 102 patients (17.59%) had a prior kidney transplant, and 24 (4.67%) were ABO incompatible. The mean cold ischemia time was 16.42 ± 13.36 h. The mean HLA-A/B/DR mismatch was 3.86 ± 1.44. The baseline characteristics of the recipients at the time of transplantation are summarized in Table 1 with the comparison of patients with stable (Kidney graft instability was defined according to the acute kidney injury 2012 KDIGO guidelines [26] as an increase of serum creatinine of more than 0.3 mg per deciliter (>26.4 μmol/L) or of more than 50% from baseline and the presence of proteinuria) and unstable kidney function. The median time between kidney transplantation and dd-cfDNA assessment was 0.39 years (IQR 0.25–1.17). At the time of dd-cfDNA measurement, the mean estimated glomerular filtration rate was 47.88 ± 20.69 mL/min/1.73 m^2^, median urinary protein-to-creatinine ratio was 0.20 g/g (IQR 0.10–0.57). At the time of dd-cfDNA measurement, 339 (56.22%) patients were clinically stable while 264 (43.78%) were unstable, with 65 (10.78%) presenting with antibody mediated rejection (AMR) and, 27 (4.48%) showing T-cell mediated rejection (TCMR) or mixed rejection. Functional, immunological, and histological characteristics at the time of dd-cfDNA evaluation are summarized in Table 2. Patients with unstable kidney allograft function showed significantly lower eGFR, higher proteinuria, and more positive anti-HLA DSA (*p* < 0.0001 for all comparisons).

**TABLE 1:** Baseline patient characteristics of the cohort according to kidney allograft stability.

	Patients (N = 580)	Stable patients (N = 324)	Unstable patients (N = 256)	*P*-value
Recipient characteristics				
Age (y), mean (SD)	51.35 (16.63)	52.11 (16.22)	50.40 (17.12)	0.257
Male, number (%)	360 (62.07%)	192 (59.26%)	168 (65.62%)	0.122
Cause of end stage renal disease				
Glomerulopathy, number (%)	149 (25.69%)	91 (28.09%)	58 (22.66%)	
Polycystic kidney disease, number (%)	64 (11.03%)	37 (11.42%)	27 (10.55%)	
Interstitial nephritis, number (%)	31 (5.34%)	19 (5.86%)	12 (4.69%)	
Diabetes, number (%)	49 (9.45%)	26 (9.02%)	23 (8.98%)	
Vascular, number (%)	60 (10.34%)	37 (11.42%)	23 (8.98%)	
Other, number (%)	86 (14.83%)	46 (14.20%)	40 (15.62%)	
Unknown etiology, number (%)	141 (24.31%)	68 (20.99%)	73 (28.52%)	0.362
Donor characteristics				
Age (y), mean (SD)	53.61 (17.50)	54.14 (16.52)	52.95 (18.67)	0.661
Male, number (%)	339 (58.45%)	189 (58.33%)	150 (58.59%)	1
Deceased donor, number (%)	399 (68.79%)	210 (64.81%)	189 (73.83%)	0.024
Transplant baseline characteristics				
Prior kidney transplant, number (%)	102 (17.59%)	52 (16.05%)	50 (19.53%)	0.275
Cold ischemia time (h), mean (SD)	16.42 (13.36)	16.47 (14.12)	16.36 (12.54)	0.528
HLA-A/B/DR mismatch, mean (SD)	3.86 (1.44)	3.85 (1.49)	3.88 (1.39)	0.365
ABO incompatible transplant, number (%)	24 (4.67%)	15 (4.98)	9 (4.23)	0.833

Abbreviations: HLA, human leucocyte antigen.

**TABLE 2:** Characteristics at the time of biopsy with concomitant decentralized and centralized dd-cfDNA measurements according to kidney allograft stability.

	Evaluations (N = 603)	Stable patients (N = 339)	Unstable patients (N = 264)	*P*-value
Time from transplant to evaluation (y), median (IQR)	0.39 (0.25–1.17)	0.27 (0.25–1.00)	1.18 (0.28–5.17)	<0.0001
Estimated GFR, mean (SD)	47.88 (20.69)	54.78 (20.05)	39.01 (17.98)	<0.0001
Proteinuria (g/g), median (IQR)	0.20 (0.10–0.57)	0.15 (0.08–0.30)	0.40 (0.14–1.08)	<0.0001
Positive anti-HLA DSAs, number (%)	304 (50.41%)	140 (41.30%)	164 (62.12%)	<0.0001
Biopsy findings				
Active AMR, number (%)	47 (7.79%)	19 (5.60%)	28 (10.61%)	
Chronic active AMR, number (%)	18 (2.99%)	2 (0.59%)	16 (6.06%)	
Inactive AMR, number (%)	3 (0.50%)	—	3 (1.14)	
Equivocal for diagnosis of AMR, number (%)	7 (1.16%)	1 (0.29%)	6 (2.27%)	
Acute TCMR, number (%)	9 (1.49%)	3 (0.88%)	6 (2.27%)	
Chronic active TCMR, number (%)	11 (1.82%)	2 (0.59%)	9 (3.41%)	
Mixed rejection, number (%)	7 (1.16%)	2 (0.59%)	5 (1.89%)	
Borderline lesions, number (%)	3 (0.50%)	2 (0.59%)	1 (0.38%)	
Viral nephritis, number (%)	12 (1.99%)	8 (2.36%)	4 (1.52%)	
Glomerulitis without rejection, number (%)	19 (3.15%)	13 (3.83%)	6 (2.27%)	
FSGS, number (%)	18 (2.99%)	2 (0.59%)	16 (6.06%)	
IF-TA, number (%)	222 (36.82%)	117 (34.51%)	105 (39.77%)	
No specific lesions, number (%)	227 (37.65%)	168 (49.56%)	59 (22.35%)	—

Abbreviations: GFR, glomerular filtration rate; HLA, human leucocyte antigen; DSA, Donor-Specific antibody; AMR, Antibody-mediated rejection; TCMR, T-Cell mediated rejection; FSGS, focal segmental glomerulosclerosis; IF-TA, interstitial fibrosis and tubular atrophy.

### Comparison of Decentralized and Centralized dd-cfDNA Results

**FIGURE 1:**
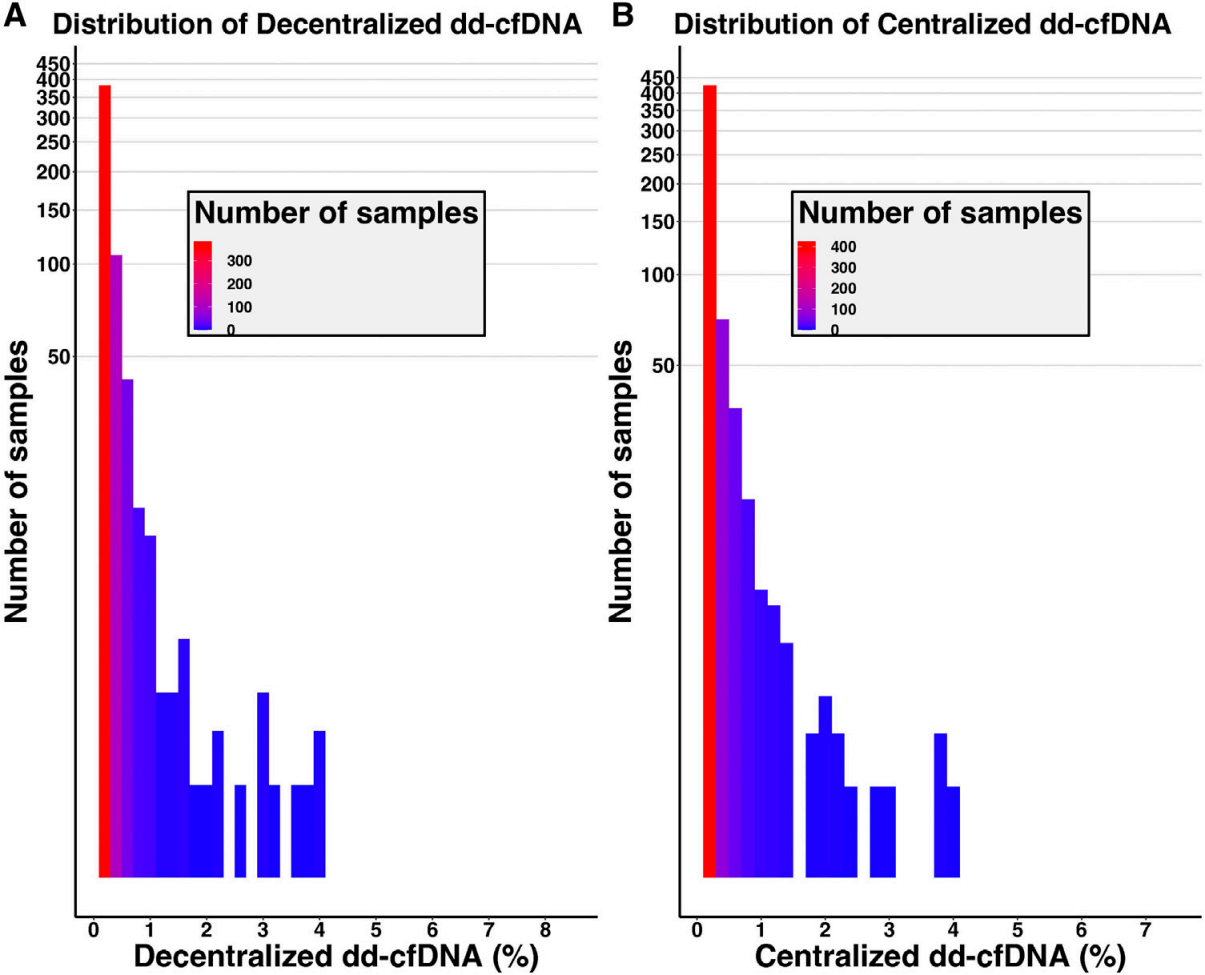
Distribution of decentralized **(A)** and centralized **(B)** dd-cfDNA results among the cohort. The y-axis corresponds to the number of samples (logarithmic scale).

**FIGURE 2:**
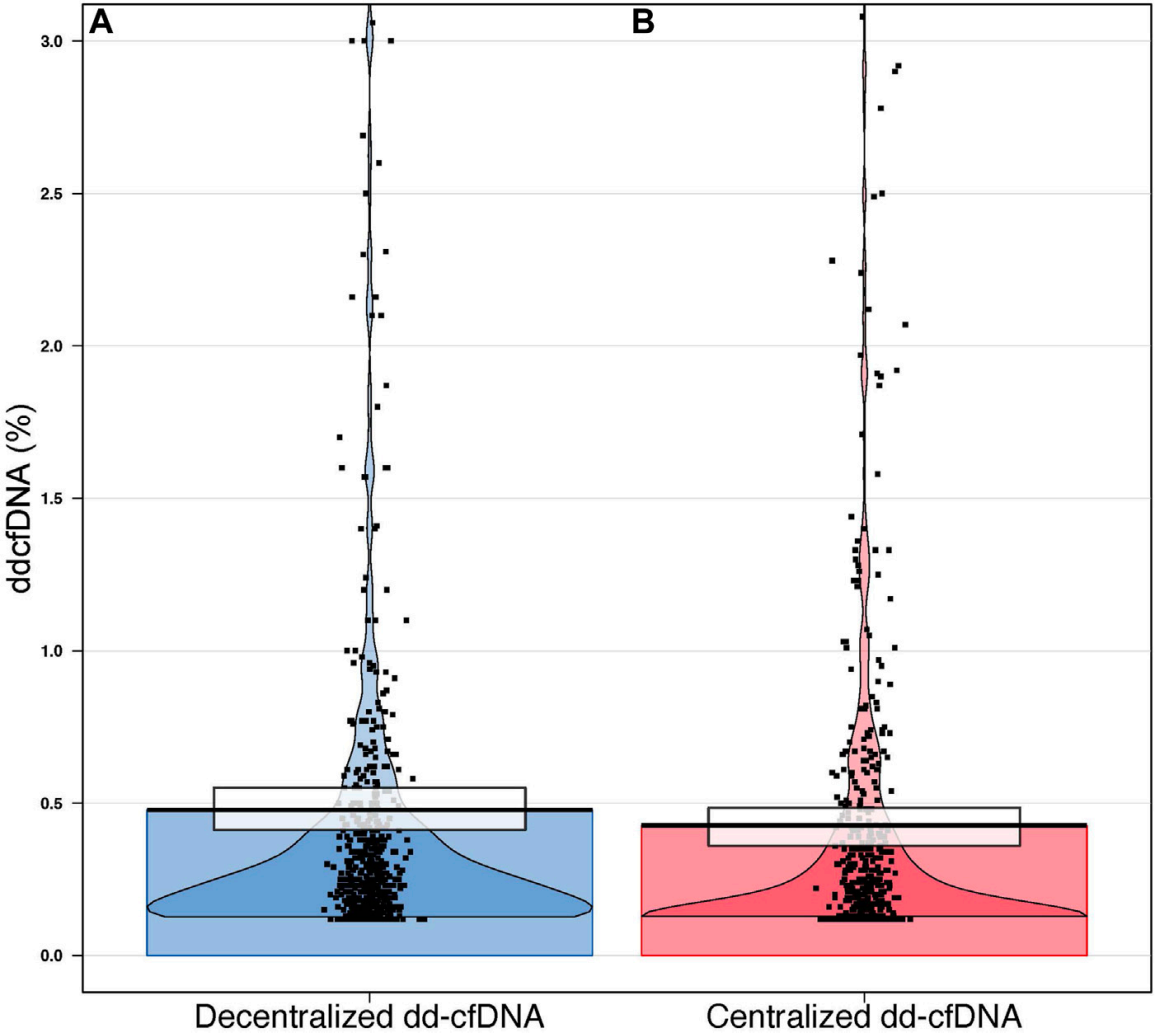
Violin plot distribution of decentralized **(A)** and centralized **(B)** dd-cfDNA results. Each point represents a single sample. The black horizontal lines represent the central tendencies. The beans represent the smoothed densities, and the rectangles represent the inference intervals with confidence intervals (decentralized dd-cfDNA in blue, centralized dd-cfDNA in red).

**FIGURE 3:**
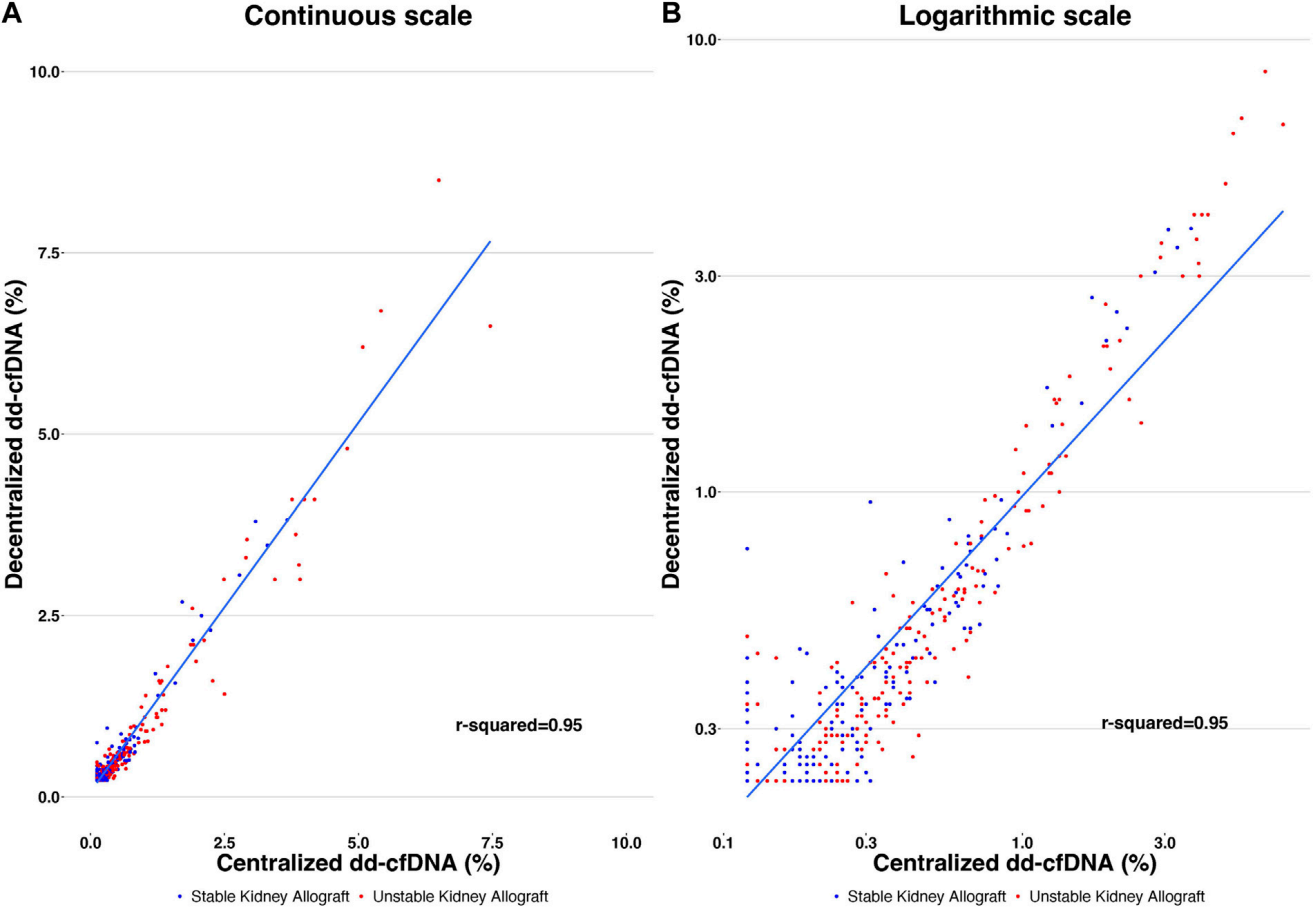
Correlation of decentralized and centralized dd-cfDNA results, with *r* ^2^ = 0.95. Each point represents a single sample. The y-axis corresponds to the decentralized dd-cfDNA and the x-axis corresponds to the centralized dd-cfDNA using continuous scale **(A)** and logarithmic scale **(B)**.

The mean dd-cfDNA levels from decentralized and centralized assays were 0.51% ± 0.81% [median: 0.23, interquartile range (IQR): 0.23–0.42] and 0.43% ± 0.78% (median: 0.17, IQR: 0.12–0.37), respectively. Figure 1 shows the distribution of decentralized and centralized dd-cfDNA results and Figure 2 shows the violin plots of the two tests. The decentralized assay showed high correlation with centralized dd-cfDNA results with *r* ^2^ = 0.95 and a Spearman’s rank correlation of 0.88 (*p* < 0.0001). Correlation between the two tests is represented in Figure 3.

### Association and Discrimination of Decentralized and Centralized dd-cfDNA Results With Rejection

**FIGURE 4:**
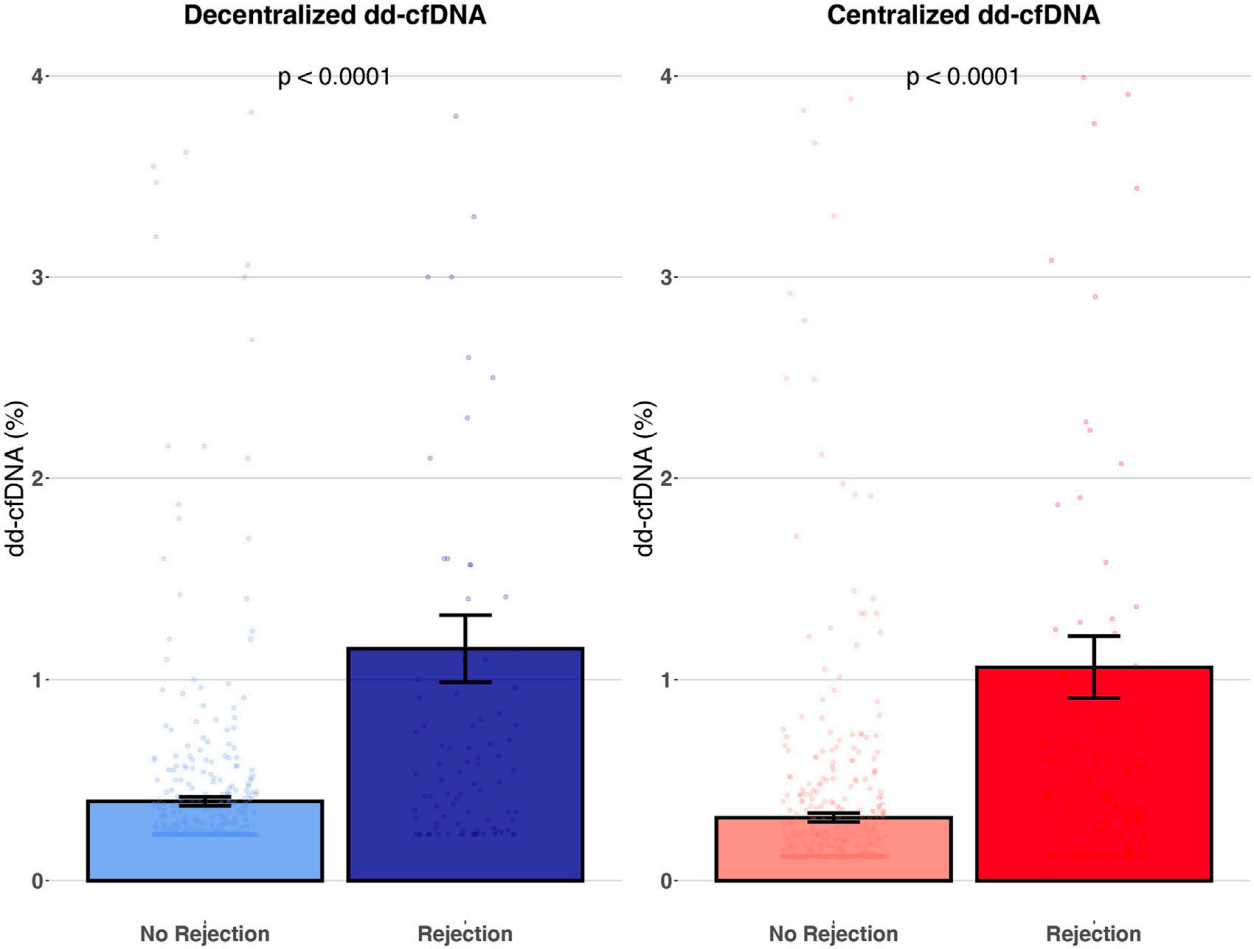
Association of decentralized and centralized dd-cfDNA with rejection. This figure represents the mean level of decentralized and centralized dd-cfDNA according to the presence or absence of rejection. Each dot corresponds to an individual dd-cfDNA value. Data are presented as mean values ± SEM.

Mean dd-cfDNA levels of the decentralized assay were 1.15% ± 1.60% (median: 0.54, IQR: 0.26–1.10) for biopsies showing rejection (AMR, TCMR and mixed rejection) and 0.39% ± 0.48% (median: 0.23, IQR: 0.23–0.36) for biopsies without rejection (*p* < 0.0001). Mean dd-cfDNA levels of the centralized assay were 1.06% ± 1.47% (median: 0.48, IQR: 0.22–1.04) for biopsies showing rejection and 0.31% ± 0.49% (median: 0.14, IQR: 0.12–0.29) for biopsies without rejection (*p* < 0.0001) (Figure 4). The decentralized and centralized assays showed strong correlation among biopsies both without concurrent allograft rejection (*r* ^2^ = 0.94) and with ongoing rejection (*r* ^2^ = 0.95).

**FIGURE 5:**
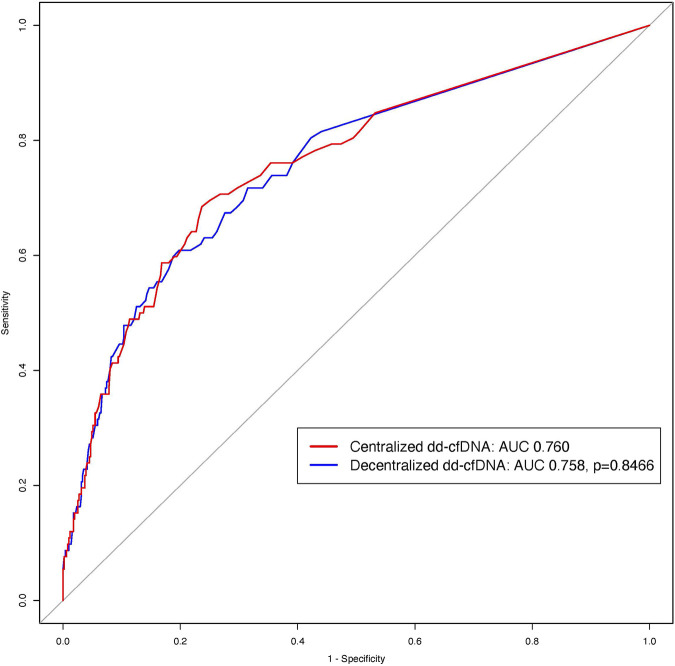
ROC curves of the centralized and decentralized dd-cfDNA to detect rejection. The red ROC curve corresponds to centralized dd-cfDNA alone and the blue curve to decentralized dd-cfDNA alone for the detection of rejection. There was no difference between the two tests (*p* = 0.8466).

Both assays showed significant and strong association with allograft rejection using a logistic regression: decentralized dd-cfDNA (log transformation) (OR = 3.293, 95% CI: 2.453–4.421; *p* < 0.0001) and centralized dd-cfDNA (OR = 2.722, 95% CI: 2.146–3.454; *p* < 0.0001). The discriminations of decentralized dd-cfDNA (log transformation) and centralized dd-cfDNA to detect rejection were similar without significant difference (AUC: 0.758 and 0.760, respectively; *p* = 0.8466) (Figure 5).

### Sensitivity Analyses

Various analyses were performed to further confirm the robust correlation between dd-cfDNA results from decentralized and centralized assays in different clinical scenarios. With respect to post-transplant timepoint of evaluation, the high correlation between decentralized and centralized dd-cfDNA results remained when assessed within (*r* ^2^ = 0.97) or beyond (*r* ^2^ = 0.94) the first year post-transplantation. Regarding allograft stability, good correlation was observed for patients with stable (*r* ^2^ = 0.95) compared to unstable (*r* ^2^ = 0.95) renal function. Among different rejection phenotypes, the good correlation was maintained for AMR (*r* ^2^ = 0.96) versus TCMR and/or mixed rejection (*r* ^2^ = 0.97).

Bland-Altman plot was assessed to visualize the differences between the decentralized and centralized dd-cfDNA measurements. The average difference was 0.08 (95% CI: 0.06–0.09), the upper limit of agreement was 0.43 (95% CI: 0.41–0.45), and the lower limit of agreement was −0.26 (95% CI: −0.24 to −0.28) (Supplementary Figure S1). A Passing-Bablok regression was performed for the comparison of the decentralized and centralized dd-cfDNA test (Supplementary Figure S2). The slope of the fitted regression was 0.76 (95% CI: 0.70–0.82) and the intercept was 0.139 (95% CI: 0.132–0.145).

## Discussion

This large, multi-national study has demonstrated the accuracy of decentralized dd-cfDNA solution AlloSeq cfDNA compared to heavily utilized centralized dd-cfDNA platform cfDNA AlloSure Kidney. The generalisability of the decentralized dd-cfDNA assay has been validated in distinct cohorts and various clinical scenarios [27–40]. This is the first large, multi-center validation study on a European cohort directly comparing the analytical correlation and clinical assay performance of these two assays.

The decentralized and centralized dd-cfDNA assays yielded good correlation overall and both tests were strongly associated with allograft rejection, including AMR and TCMR and/or mixed rejection. Results were also consistent across several clinical scenarios including the interval between transplantation and the timing of dd-cfDNA evaluation, in stable and unstable patients, further highlighting the robustness and value of this decentralized dd-cfDNA assay. However, the correlation of these two assays and their associations with other situations, such as BK virus associated nephropathy, urinary tract infection, or sepsis, were not assessed in this study because no association was shown in a previous study [13].

The observed slope of 0.76 for the correlation between decentralized and centralized dd-cfDNA results indicates that the centralized dd-cfDNA method tends to provide lower values compared to the decentralized method, particularly at higher levels. However, values exceeding 1% represent a threshold beyond which the probability of rejection is high [9]. Therefore, the lack of agreement at these higher values is of limited clinical concern, as any results in that range from either method would lead to further diagnostic actions, such as a biopsy, regardless of the discrepancy.

dd-cfDNA testing is an efficient, informative, and minimally invasive solution for post-transplant monitoring in kidney transplant patients [13]. Implementation of this biomarker for routine surveillance to inform on potential injury or rejection enables clinicians to more promptly modify immunosuppressive treatment [41–43]. The predicate centralized dd-cfDNA testing platform is well-characterized and broadly used for solid organ transplant patients in the United States [9–12]. The decentralized dd-cfDNA assay evaluated here is a commercial kit with international availability and support, offering an efficient solution for decentralized dd-cfDNA quantitation. Both assays share the same fundamental biochemistry. Curated panels of SNP loci are amplified via PCR, then resulting amplicons are indexed using NGS barcodes, pooled, and purified in preparation for NGS. The decentralized dd-cfDNA assay targets 202 SNPs, performs amplification and indexing in a single PCR, and has been validated with Illumina MiSeq and MiniSeq, while the centralized dd-cfDNA assay targets 405 SNPs, requires two independent PCR steps, and uses Illumina NextSeq [24].

These decentralized and centralized dd-cfDNA assays have been compared in internal manufacturer studies, yielding strong concordance with *r* ^2^ = 0.9136 for clinical samples, *r* ^2^ = 0.9458 for analytical samples near the limit of detection, and *r* ^2^ = 0.9991 in the range 1%–70% for linearity (unpublished manufacturer data). With respect to other dd-cfDNA testing methods, various studies have revealed that this decentralized dd-cfDNA assay was well-correlated with digital droplet PCR (ddPCR) [39], quantitative fluorescent PCR (QF-PCR) amplification of short tandem repeats (STR) [31], and high-throughput sequencing [33], with higher sensitivity than both ddPCR [39] and QF-PCR [31]. Several other recent studies have also demonstrated the use of this decentralized dd-cfDNA assay for kidney transplant monitoring [27–30, 32, 34–38, 40].

The results from this study demonstrate the accuracy of this decentralized dd-cfDNA assay with respect to the predicate centralized dd-cfDNA assay. This decentralized assay leverages low input, NGS sensitivity, and associated analysis software to yield accurate dd-cfDNA results, offering increased convenience in clinical practice compared to centralized assays. Onsite testing would allow transplant centers flexibility in both throughput and testing schedule, enabling implementation for not only standard patient monitoring but also clinical trials. However, further studies are needed with the comparisons between the two assays regarding the efficiency in terms of turnaround time and cost. The utilization of decentralized dd-cfDNA assays, such as the one evaluated here, addresses the current need for a powerful and efficient tool to expand access and broaden adoption of dd-cfDNA testing for kidney transplant surveillance.

## Conclusion

The decentralized dd-cfDNA assay evaluated in this study shows strong correlation with the well-characterized and broadly used centralized dd-cfDNA assay. Though this behaviour cannot be generalized to other assays or methods without further study, the good concordance demonstrated here illustrates the potential of decentralized dd-cfDNA testing. Moreover, owing to its high accuracy to detect rejection, this decentralized dd-cfDNA assay proves to be a significant asset in clinical practice to enhance monitoring and care of kidney transplant patients.

## Data Availability

The raw data supporting the conclusions of this article will be made available by the authors, without undue reservation.
