# Supplementary material for: Evaluation of a Decentralized Donor-Derived Cell-Free DNA Assay for Kidney Allograft Rejection Monitoring
Source: Transpl Int. 2024 Dec 17;37:13919. doi: 10.3389/ti.2024.13919 (PMC11685011; doi:10.3389/ti.2024.13919)

SUPPLEMENTAL DATA

Table S1. Decentralized dd-cfDNA assay SNP chromosome distribution.

SNP ID	Chromosome location	Reference allele frequency	SNP ID	Chromosome location	Reference allele frequency
s01001	1p36.31	0.480	s07004	7p12.3	0.492
s01002	1p31.2	0.492	s07005	7q22.2	0.526
s01003	1p21.1	0.538	s07006	7q31.2	0.473
s01004	1p13.3	0.507	s07007	7q35	0.536
s01005	1p13.2	0.523	s07008	7q36.3	0.531
s01006	1p12	0.534	s08001	8p21.2	0.457
s01007	1q41	0.532	s08002	8p21.2	0.484
s01008	1q43	0.470	s08003	8p21.1	0.481
s02001	2p25.3	0.503	s08004	8p21.1	0.525
s02002	2p24.1	0.491	s08005	8p11.22	0.486
s02003	2p24.1	0.507	s08006	8q12.1	0.505
s02004	2p23.3	0.483	s08007	8q21.11	0.487
s02005	2p22.2	0.490	s08008	8q23.1	0.489
s02006	2p22.1	0.505	s08009	8q24.21	0.467
s02007	2p21	0.525	s09001	9p24.3	0.468
s02008	2p16.3	0.458	s09002	9p22.3	0.508
s02009	2p16.2	0.533	s09003	9p21.3	0.529
s02010	2p14	0.504	s09004	9p21.1	0.541
s02011	2q12.1	0.540	s09005	9q21.13	0.487
s02012	2q12.2	0.470	s09006	9q21.13	0.486
s02013	2q14.1	0.514	s09007	9q21.13	0.512
s02014	2q21.2	0.500	s09008	9q31.3	0.497
s02015	2q22.3	0.474	s09009	9q32	0.477
s02016	2q22.3	0.501	s09010	9q32	0.512
s02017	2q23.3	0.517	s09011	9q33.3	0.512
s02018	2q31.1	0.470	s09012	9q34.11	0.489
s02019	2q32.1	0.545	s10001	10p14	0.504
s02020	2q33.3	0.500	s10002	10p13	0.483
s02021	2q35	0.494	s10003	10q22.1	0.543
s02022	2q37.3	0.479	s10004	10q23.31	0.493
s03001	3p26.1	0.506	s10005	10q24.1	0.471
s03002	3p24.1	0.495	s10006	10q25.3	0.455
s03003	3p22.3	0.523	s10007	10q26.3	0.549
s03004	3p22.3	0.546	s11001	11p15.1	0.489
s03005	3p22.1	0.494	s11002	11p13	0.458
s03006	3P14.2	0.525	s11003	11q13.5	0.468
s03007	3p13	0.507	s11004	11q14.1	0.528
s03008	3q11.2	0.508	s11005	11q14.2	0.462
s03009	3q12.1	0.508	s11006	11q14.2	0.481
s03010	3q13.2	0.498	s11007	11q23.3	0.505
s03011	3q13.31	0.484	s11008	11q23.3	0.496

s03012	3q21.3	0.502	s11009	11q24.1	0.505
s03013	3q22.1	0.494	s11010	11q24.3	0.489
s03014	3q22.1	0.543	s12001	12p13.33	0.520
s03015	3q24	0.488	s12002	12p13.32	0.544
s03016	3q24	0.497	s12003	12q14.2	0.531
s03017	3q25.1	0.538	s12004	12q22	0.516
s03018	3q25.1	0.470	s12005	12q23.3	0.529
s03019	3q25.2	0.525	s12006	12q24.22	0.498
s03020	3q25.2	0.543	s12007	12q24.23	0.485
s03021	3q26.31	0.462	s12008	12q24.31	0.519
s03022	3q27.3	0.457	s12009	12q24.32	0.466
s03023	3q28	0.458	s13001	13q13.3	0.485
s04001	4p16.2	0.492	s13002	13q21.2	0.523
s04002	4p15.31	0.538	s13003	13q21.32	0.519
s04003	4p15.1	0.519	s13004	13q21.33	0.512
s04004	4p14	0.512	s13005	13q31.3	0.517
s04005	4q12	0.544	s13006	13q33.1	0.505
s04006	4q26	0.491	s13007	13q33.3	0.488
s04007	4q28.3	0.508	s14001	14q11.2	0.534
s04008	4q31.21	0.508	s14002	14q13.1	0.494
s04009	4q31.3	0.505	s14003	14q13.1	0.475
s04010	4q32.2	0.507	s14004	14q13.2	0.503
s04011	4q35.1	0.513	s14005	14q24.3	0.494
s04012	4q35.1	0.484	s14006	14q32.11	0.475
s05001	5p13.3	0.530	s14007	14q32.13	0.530
s05002	5p13.3	0.545	s14008	14q32.2	0.489
s05003	5p13.3	0.464	s15001	15q14	0.528
s05004	5q11.2	0.531	s15002	15q14	0.483
s05005	5q11.2	0.465	s15003	15q21.2	0.498
s05006	5q13.1	0.534	s15004	15q22.2	0.527
s05007	5q14.1	0.492	s15005	15q26.1	0.499
s05008	5q21.1	0.468	s15006	15q26.1	0.549
s05009	5q21.1	0.495	s16001	16q12.1	0.528
s05010	5q21.3	0.503	s16002	16q21	0.505
s05011	5q22.3	0.467	s17001	17p13.3	0.539
s05012	5q23.1	0.520	s17002	17p12	0.463
s05013	5q31.3	0.473	s17003	17p12	0.534
s05014	5q33.1	0.530	s17004	17q12	0.471
s05015	5q34	0.528	s17005	17q23.2	0.465
s05016	5q34	0.520	s18001	18p11.31	0.462
s06001	6p25.1	0.463	s18002	18p11.22	0.475
s06002	6p21.2	0.473	s18003	18p11.22	0.499
s06003	6p21.2	0.509	s18004	18q11.2	0.482
s06004	6q13	0.516	s18005	18q21.1	0.499
s06005	6q13	0.539	s18006	18q21.31	0.493
s06006	6q14.1	0.481	s19001	19p13.2	0.544
s06007	6q22.1	0.529	s19002	19q12	0.504
s06008	6q22.31	0.498	s19003	19q12	0.528
s06009	6q22.31	0.514	s19004	19q13.31	0.504

s06010	6q22.33	0.490	s20001	20p12.1	0.535
s06011	6q22.33	0.541	s20002	20p11.22	0.475
s06012	6q22.33	0.510	s20003	20q11.23	0.454
s06013	6q23.3	0.468	s20004	20q13.12	0.525
s06014	6q25.3	0.470	s20005	20q13.2	0.542
s06015	6q25.3	0.497	s20006	20q13.2	0.505
s06016	6q25.3	0.540	s21001	21q21.3	0.480
s06017	6q26	0.508	s21002	21q22.11	0.467
s07001	7p22.3	0.535	s21003	21q22.3	0.475
s07002	7p21.1	0.474	s22001	22q13.32	0.467
s07003	7p14.3	0.523	s22002	22q13.33	0.483

Figure S1. Bland-Altman plot visualizing the differences between the decentralized and centralized dd-cfDNA Measurements. The x-axis of the plot displays the average measurement of the two tests and the y-axis displays the difference in measurements between the two tests. The central line corresponds to the average difference in measurements between the two tests. The upper line corresponds to the upper limit of the 95% CI for the average difference and the lower line to the lower limit of the 95% CI. The average difference is 0.08 (95% CI 0.06 – 0.09). The upper limit of agreement is 0.43 (95% CI 0.41 – 0.45) and the lower limit of agreement is -0.26 (95% CI -0.24 – -0.28).

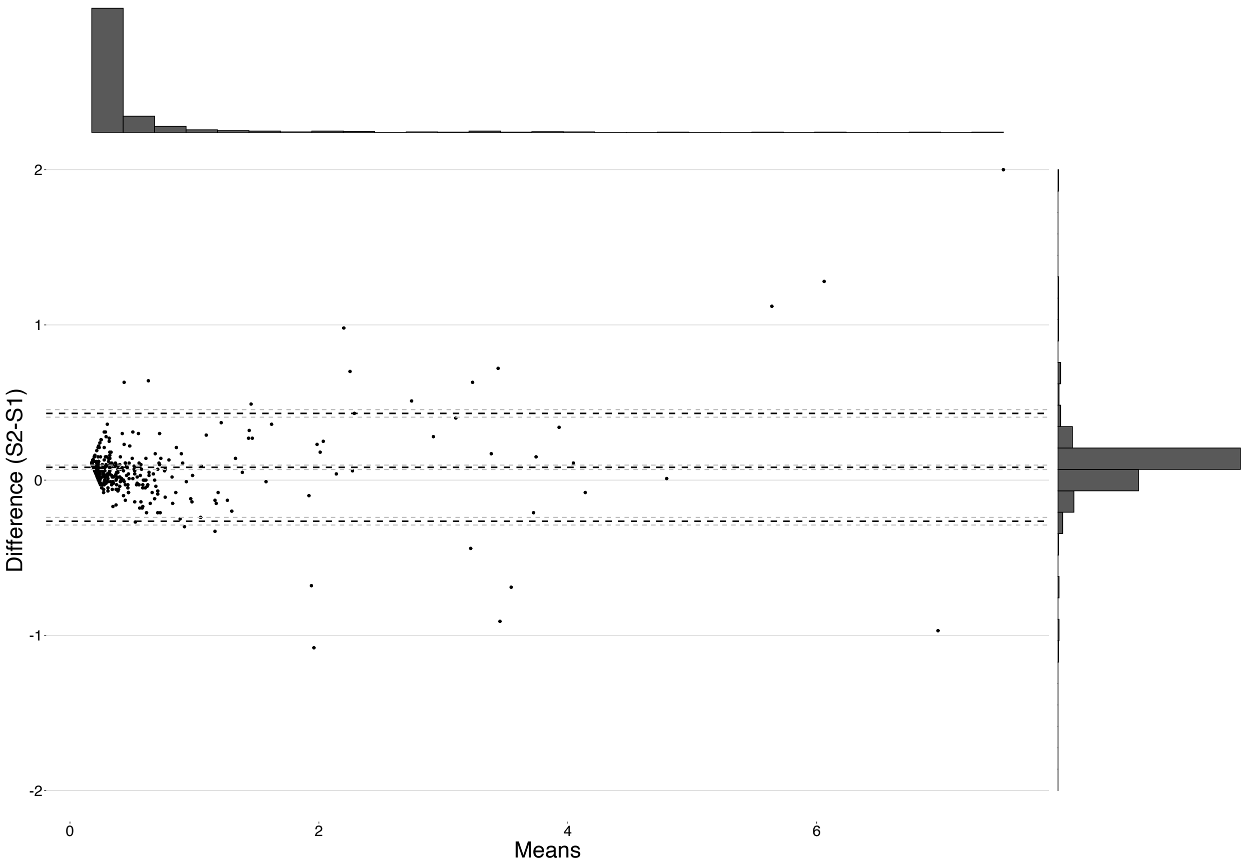

Figure S2. Passing-Bablok regression for the comparison of the decentralized and centralized dd-cfDNA results. The dashed red line corresponds to the line of identity (centralized dd-cfDNA = decentralized dd-cfDNA) and the blue line corresponds to the fitted regression with its 95% CI. The slope was 0.76 (95% CI 0.70 – 0.82), the intercept was 0.139 (95% CI 0.132 – 0.145), and the Pearson's r was 0.976.

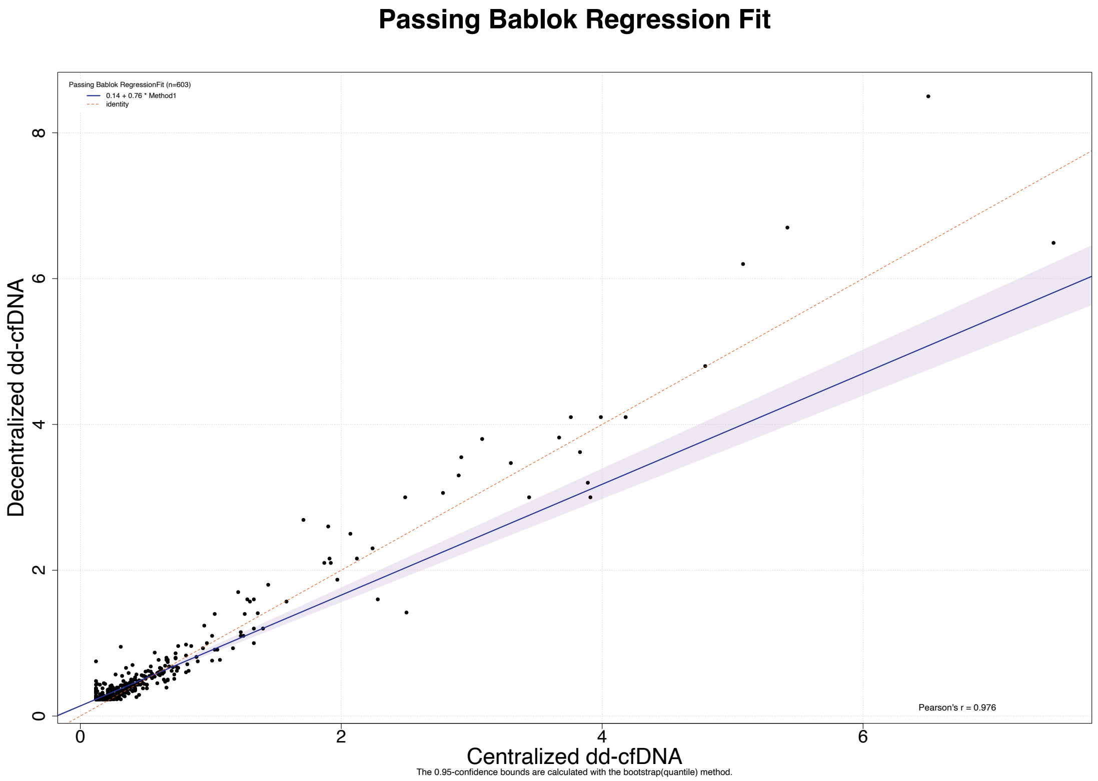

Supplement: Supplementary file 1 [file DataSheet1.PDF]
